# The absence of data on driving under the influence of alcohol in road traffic studies: a scoping review of non-randomized studies with vote counting based on the direction of effects of alcohol policies

**DOI:** 10.1186/s13011-023-00553-y

**Published:** 2023-07-28

**Authors:** Pablo Martínez, Junon Joseph, José Ignacio Nazif-Munoz

**Affiliations:** 1 Faculté de médecine et des sciences de la santé, Université de Sherbrooke, 150, Place Charles-Le Moyne, Longueuil, Québec J4K A08 Canada; 2 Centre de recherche Charles-Le Moyne—Saguenay-Lac-Saint-Jean sur les innovations en santé (CR-CSIS), 150, Place Charles-Le Moyne, Longueuil, Québec J4K A08 Canada; 3 Institut universitaire sur les dépendances, 950 Rue de Louvain Est, Montréal, Québec H2M 2E8 Canada

**Keywords:** Driving under the influence, Alcohol drinking, Legislation & jurisprudence, Traffic accidents, Systematic review, Non-randomized controlled trials

## Abstract

**Background:**

Data on driving under the influence of alcohol (DUIA) are not always available, accurate, or reliable, making it difficult to study the effects of alcohol policies on road traffic outcomes. The objectives of our study were twofold: 1) to describe how road traffic outcomes of alcohol policies are assessed when DUIA data are missing, and 2) to explore the effects of alcohol policies when DUIA data are missing.

**Methods:**

We conducted a scoping review of non-randomized studies that assessed the road traffic outcomes of alcohol policies when DUIA data are missing. Until November 2021, we searched studies published between 2000 and 2021, in English or French, via MEDLINE, APA PsycInfo, CINAHL, and SocINDEX. We assessed the risk of bias in the included studies with the Quality Assessment Tool for Before-After (Pre-Post) Studies With No Control Group. The selection process, data extraction, and the risk of bias assessment were conducted independently and in duplicate. We used vote counting based on the direction of the effects of alcohol policies as a synthesis method. The protocol for this review was published in PROSPERO under record number CRD42021266744.

**Results:**

Twenty-four eligible studies were included. Regarding objective 1, most studies used uncontrolled interrupted time series designs to assess road traffic fatalities resulting from night-time crashes. The reasons for missing DUIA data were generally not reported. Regarding objective 2, we found evidence for an association between alcohol policies and decreased road traffic fatalities. Subgroup analyses found no evidence for an association between methodological modifiers and positive effect directions for road traffic fatalities.

**Conclusion:**

Caution is needed when interpreting road traffic outcomes associated with alcohol policies when DUIA data are missing. Greater efforts should be made to improve the reporting of outcomes assessments. Future studies must address several methodological issues (e.g., more granular data, well-defined intervention and implementation, and controlled designs). Our results should be compared to those from others reviews where DUIA data were available to confirm or recalibrate the associations found in studies where DUIA data were missing.

**Supplementary Information:**

The online version contains supplementary material available at 10.1186/s13011-023-00553-y.

## Introduction

Road traffic crashes, injuries, and deaths have serious public health and economic consequences affecting sustainable development [1, 2]. According to the most recent analyses for the Global Burden of Diseases, Injuries, and Risk Factors Study (GBD), road traffic injuries were responsible for more than 900,000 deaths in 2019 and are currently among the top causes of disability-adjusted life years, mainly affecting the male population, adolescents (10 to 24 years) and adults (25 to 49 years) [3].

Acute alcohol consumption, even at small doses, has been consistently associated with poor driving performance [4] and increased risk for road traffic crashes, injuries, and deaths [5, 6]. Alcohol consumption is one of the main contributors to adverse road traffic outcomes. Borges et al. [7], using the GBD 2019 data, found that 6.6% of all road traffic injuries in 2019 were attributable to alcohol consumption. The World Health Organization (WHO) estimated that alcohol consumption accounted for 27% of road traffic deaths worldwide [8].

Given the severe consequences of driving under the influence of alcohol (DUIA), the WHO has consistently urged member states to advance and enforce countermeasures explicitly aimed at reducing DUIA, such as low blood alcohol concentration (BAC) limits and sobriety checkpoints [9, 10]. These measures have been comprehensively studied and are mainly focused on increasing the perceived risk of detection and punishment [11–13]. A recent overview of systematic reviews concluded that sobriety checkpoints and random breath testing were consistently found to reduce alcohol-related road traffic crashes [14]. However, a broad range of strategies may reduce the problem of DUIA [12]. There is evidence that general alcohol policies restricting access to alcohol (e.g., alcohol taxation and minimum legal drinking age laws) may indirectly reduce road traffic deaths by decreasing high-risk alcohol consumption and DUIA [11, 15–18].

Notwithstanding, the impact assessment of these strategies is severely limited by the availability and reliability of road safety data, being prone to errors and inconsistencies [19–22]. Likewise, the legal and technical capabilities for DUIA enforcement vary considerably between and within countries [23]. The challenges around road safety data and DUIA enforcement particularly disadvantage low- and middle-income countries (LMIC), those most affected by the burden of road traffic injuries and fatalities [23–25]. In some high-income countries, objective measurement of DUIA occurs sporadically and is not always registered in police reports [12, 20]. Thus, studies had to rely on surrogate measures (e.g., night-time fatal crashes), assuming a higher probability of alcohol involvement for these traffic crashes [12].

The objectives of our review were twofold: 1) to describe how traffic crashes outcomes of alcohol policies are assessed when DUIA data are missing, and 2) to explore the effects of alcohol policies when DUIA data are missing. The potential contribution of our study is relevant and timely. The descriptive approach may suggest alternative research designs, and easily accessible and reliable surrogate measures in settings where DUIA data may be challenging to obtain – especially in LMIC. The exploratory evaluation of alcohol policies – which we have broadly categorized as general and specific – and their effects on traffic outcomes – i.e., traffic crashes, injuries, and fatalities – is a critical application for surrogate measures of DUIA data.

## Material and methods

### Study design

We conducted a scoping review with vote counting based on the direction of effect, combining a descriptive with an exploratory synthetic approach. We reported our study per the Preferred Reporting Items for Systematic Reviews and Meta-Analyses (PRISMA) statement [26]. The protocol for this study was registered prospectively in the International Prospective Register of Systematic Reviews (PROSPERO) under registration number CRD42021266744 [27]. All protocol updates are detailed in Additional file 1: Appendix 1.

### Eligibility criteria

We followed the Population, Intervention, Comparator, Outcomes, and Study Design (PICOS) framework to define eligibility criteria at the study level [26]. Studies had the following characteristics to be eligible:

- *Population:* All road traffic users (i.e., passengers, pedestrians, and drivers of motorized or non-motorized vehicles). We did not exclude any population based on age, sex, ethnicity, or other characteristics.
- *Intervention:* An intervention condition in which the population was exposed to an alcohol policy. Alcohol policies were included based on their expected direct and indirect effects on DUIA. We followed the classification implied by Shults et al. [12] in which general (e.g., banning alcohol production and sale without a license) and specific (e.g., sobriety checkpoints) alcohol policies can be found. We included studies testing a combination of different alcohol policies.
- *Comparator:* A control condition in which the population has not been exposed to the alcohol policies studied or acted as its own control (i.e., pre-intervention period). Studies with an active control condition (i.e., other alcohol policy) were also included.
- *Outcomes:* Traffic crashes, injuries, and fatalities. These outcomes could be expressed as counts or rates per population. We excluded studies that reported DUIA data.
- *Study design:* We followed the definition by Reeves et al. [28] of non-randomized studies: "any quantitative study estimating the effectiveness of an intervention (benefit or harm) that does not use randomization to allocate units to comparison groups". We excluded randomized controlled trials. Although labeling of non-randomized studies varies widely, examples of studies considered for inclusion were interrupted time series (either controlled or not), before-and-after studies (either controlled or not), retrospective or prospective cohort studies, case–control studies, and cross-sectional studies [29]. We excluded case studies and case series.

To be included, reports must have been published from 2000 to 2021. Searches were limited to reports published in English or French. Publication types not containing original results (e.g., editorials, systematic reviews, and meta-analyses) were excluded.

### Information sources

We searched the MEDLINE, APA PyscInfo, CINAHL, and SocINDEX databases via EBSCOhost from inception to the present. We also searched the reference lists of included articles for additional eligible publications. The date when each source was last searched or consulted was November 2021. We conducted an updated MEDLINE search of reports published between November 2021 and April 2023 (Additional file 1: Appendix 1).

### Search strategy

We used a simple and broad search strategy based on free-text words to maximize search results related to the intersection between DUIA and traffic outcomes (i.e., "alcohol" and "traffic crashes"). No filters or limiters were used except for the language limiter. The detailed rationale for the search strategy and its comparison with a more complex search strategy is provided in Additional file 1: Appendix 1.

### Selection process

All the records identified through the search process were extracted and imported into an Excel spreadsheet. Duplicate records were manually removed, titles and abstracts were screened, and full-text reports were retrieved and assessed for eligibility. Reports were examined to avoid duplication of data from the same study. JJ and PM conducted the selection process through independent and duplicate reviews. Disagreements were solved through discussion and the involvement of a third reviewer (JINM) who supervised the reviewing process.

### Data collection process

More than one reviewer collected data from each report. First, the reports were distributed between JJ and PM for non-duplicate data extraction. Then, PM and JINM jointly conducted a final quality assurance check. Disagreements in data collection were solved through discussion between PM and JINM. We did not confirm data from study investigators or use automation tools during this process, and we did not find multiple reports of a single study.

### Data items

The data items extracted were:

- *Report characteristics*: Leading author, year of publication, complete reference, funding source, and declared conflicts of interest.
- *Population*: Type of road traffic user (i.e., passengers, pedestrians, and drivers of motorized or non-motorized vehicles), mean age (or age range), sex (proportion female), and geographical location. We assumed the data referred to all road users if road traffic users were not specified. When information on drivers' age was unavailable, we assumed it referred to drivers aged 15 or older.
- *Intervention*: Content of the alcohol policy evaluated, including implementation dates and places (i.e., national or sub-national level). A categorization of alcohol policies into general (i.e., measures restricting access to alcohol that may indirectly reduce road traffic deaths), specific (i.e., measures explicitly aimed at reducing DUIA), or mixed was conducted per their expected impact on DUIA.
- *Comparator*: Whether the comparator was a no-intervention control condition, the population acted as its own control (e.g., a pre-intervention period) or an active control condition (e.g., another alcohol policy). If another alcohol policy was considered a comparator, we extracted data as in the *intervention*.
- *Outcomes:* Type of road traffic outcome (i.e., crashes, injuries, and fatalities), type of road traffic crash (e.g., single-vehicle collisions, intersection collisions, or collisions involving pedestrians and cyclists), and temporal information on road traffic crashes (e.g., time of day and day of the week). Additionally, we considered the time interval between data points (e.g., daily, monthly, annually), the data collection period, the outcome data source (e.g., the national or sub-national institutions providing access to data), and the rationale provided by the study authors for not using alcohol-related crash data.
- *Study design*: We distinguished between before-and-after studies and interrupted time series. Before-and-after studies conducted pooled analyses with a small number of measurements for the difference between the pre-and post-intervention periods, whereas interrupted time series considered at least two pre- and post-intervention measurements to account for changes over time and pre-intervention trends [30]. We added the "controlled" prefix when an external comparison group was used.
- *Study results*: We anticipated various effect measures reported in the included studies. Counts or rates of the selected road traffic outcomes might be analyzed as rate ratios, differences in rates, or mean differences [31]. These effect measures were included with their available measures of uncertainty (e.g., 95% confidence intervals, standard errors, or *P*-values). Outcomes were treated differently and analyzed using different techniques across studies. Therefore, we did not consider the effect measures suitable for synthesis, and no data transformation was conducted [32]. Data were extracted on the study's overall conclusion in a synthesized statement on the association between alcohol policies and road traffic outcomes (i.e., the direction of effect).

### Study risk of bias assessment

We assessed the risk of bias (RoB) in the included studies using the Quality Assessment Tool for Before-After (Pre-Post) Studies With No Control Group (abbreviated QAT-BA) developed by the National Heart, Lung, and Blood Institute [33]. The QAT-BA is a simple, generic quality and RoB assessment for before-and-after studies (including time series). The QAT-BA explores twelve domains 1) study question; 2) eligibility criteria and study population; 3) representativeness of study participants; 4) participants' enrollment; 5) sample size; 6) description and implementation of the intervention; 7) outcomes measures; 8) blinding of outcome assessors; 9) follow-up rate; 10) statistical analysis; 11) multiple outcome measures; 12) group-level interventions and individual-level outcome efforts. The domains related to blinding of outcome assessors, follow-up rate, and use of individual-level data did not apply to the RoB assessment, as all of the included studies used administrative datasets and worked (mostly) with pooled time-series cross-sectional data. We used the rule of thumb of 50 units of time as the minimum sample size required for a well-powered time series analysis in road safety studies [34]. The RoB assessment provided a binary score for each QAT-BA's domains: "positive" for low risk or "negative" for some concern for bias. The QAT-BA does not provide a cut-off score for a general classification of studies according to their RoB. The tool's authors warn against using specific rules or adding-up items for the critical appraisal of studies [33]. The RoB assessment was conducted by PM and Lysiane Robidoux with the supervision of JINM. We did not obtain or confirm relevant information from study investigators or use automation tools during this process.

### Synthesis methods

As we found substantial variation in the populations, the interventions, the comparators, the outcomes, and the study designs, we did not conduct a meta-analysis and reported our synthesis approach following the Synthesis Without Meta-analysis (SWiM) guideline [35]. We synthesized study characteristics and RoB assessment in included studies using a tabular format. Study characteristics were reported regardless of overlap with other included primary studies (e.g., two studies in the same place with overlapping years).

Our synthesis method was vote counting based on the direction of effect, regardless of statistical significance. We did not consider the statistical significance of effects as attention to this feature may lead to the exclusion of underpowered studies [32]. Owing to the highly heterogeneous study characteristics (such as the lack of a consistent effect measure), we relied on the direction of effects as a standardized binary metric [32]. Each effect estimate was categorized as positive or negative based on the observed direction of effect for road traffic outcomes. Road traffic outcomes showing a decrease were considered "positive" effect estimates (e.g., decreases in road traffic fatalities were judged to be desirable health outcomes), whereas increases in these outcomes were considered "negative" effect estimates (i.e., they represented undesirable health outcomes).

To implement vote counting based on the direction of effect, we followed the study-level step-by-step procedures detailed by Boon and Thomson [36]:

1. We grouped traffic outcomes into crashes, injuries, and fatalities.
2. We counted positive and negative effect estimates per each type of traffic outcome.
3. A positive or negative direction of effect was assumed if the vast majority of effect estimates (i.e., 70% or more) of a traffic outcome within a study followed a positive or negative direction of the effect, respectively.
4. An unclear direction of effect was assigned in those cases where a consistent direction of effect was not found within a study (i.e., less than 70% of effect estimates were in the same direction).

A summary of the study-level findings for traffic crashes, injuries, and fatalities was presented through effect direction plots [36]. We implemented a binomial probability test (setting the significance level at a *P-*value < 0.05) in Stata 16, to synthesize effect directions across studies, formally evaluating whether there was sufficient evidence of an effect (i.e., alcohol policies being associated with a positive or negative effect direction for a particular traffic outcome) [32]. The binomial probability test did not include studies with unclear effect directions [36].

We conducted subgroup analyses to test the effects of potential methodological modifiers. Firstly, we stratified studies by type of alcohol policy (i.e., general or specific), type of road traffic crash, and temporal information on road traffic crashes. Then, we implemented vote counting based on the direction of effect [36]. Finally, Fisher's exact test was used to explore whether the proportion of studies reporting positive or negative directions of the effect differed between the strata of the methodological modifiers considered.

Given the substantial variation in the attributes of included studies, we did not conduct a non-reporting bias assessment in our review nor provided a formal assessment of the certainty of our synthesis findings.

## Results

### Study selection

**Fig. 1:**
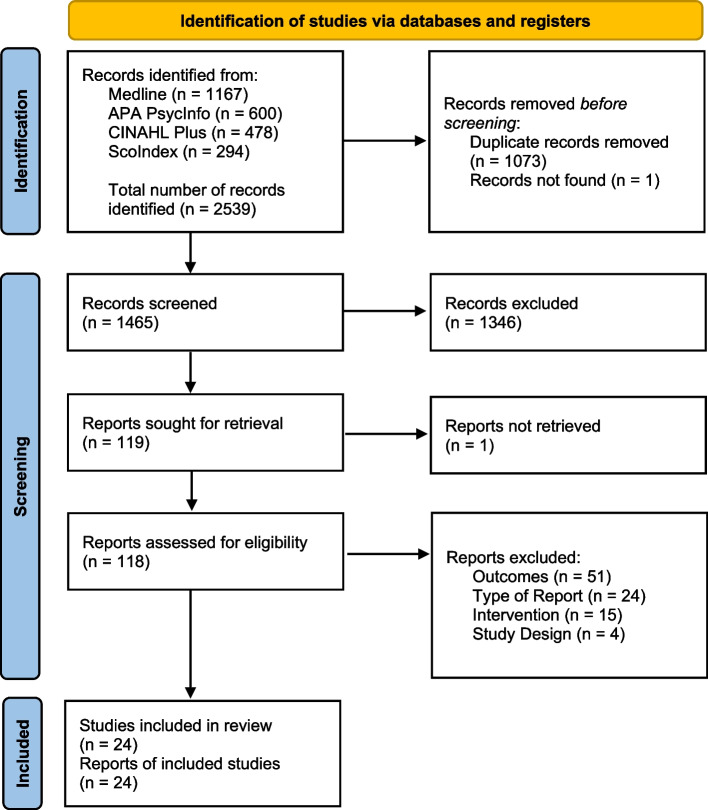
PRISMA 2020 flow diagram

We identified a total of 2,539 records from database searching. After duplicate removal, we screened 1,465 records and retrieved 118 full-text reports. The assessment of eligibility criteria led to the final inclusion of 24 studies [37–60]. The reasons for excluding 94 full-text reports were listed, with the main reason being the presence of alcohol-related data for the outcomes. We found no reviews addressing the same research question. The PRISMA 2020 flow diagram was used to report the selection process (Fig. 1).

### Study characteristics

The characteristics of the 24 included studies are summarized in Table 1. Population characteristics (i.e., type of road traffic user, age, and sex) were often not reported [37, 38, 41–44, 46, 47, 49–52, 55–60]. Studies that reported data typically considered drivers aged 15 years or older.

**Table 1:** Characteristics of included studies

Author, Year	Population	Intervention	Comparator	Outcome	Study Design
Andreuccetti et al., 2011 [37]	Drivers, passengers, and pedestrians aged 15 years or older	Reduction of legal BAC to 0.2 g/l in Brazil (2008) (specific alcohol policy)	Not external, pre-intervention. Legal BAC of 0.6 g/l (specific alcohol policy)	Monthly rates (per population) of crashes resulting in injuries and fatalities from 2001 to 2010	ITS
Assum, 2010 [38]	Drivers aged 15 years or older	Reduction of legal BAC to 0.2 g/l in Norway (2001) (specific alcohol policy)	Not external, pre-intervention. Legal BAC of 0.5 g/l (specific alcohol policy)	Annual percentages of single-vehicle, night-time, and weekend crashes resulting in injuries and fatalities from 1996 to 2005	BA
Bernat et al., 2004 [39]	Drivers aged 21 years or older	Reduction of legal BAC to 0.8 g/l in 19 USA jurisdictions prior to 2001 (specific alcohol policy)	Not external, pre-intervention. Legal BAC of 1.0 g/l in 17 of the 19 USA jurisdictions (specific alcohol policy)	Monthly rates (per population) of single-vehicle, night-time crashes resulting in fatalities from 2000 to 2012	ITS
Brubacher et al., 2017 [40]	Drivers, 29.1% aged 20–39 years, 52.0% male	Immediate roadside penalties for DUIA in B.C., Canada (2010) (specific alcohol policy)	Not external, pre-intervention. Claims for all crashes (control series). Previous administrative sanctions for DUIA (specific alcohol policy)	Monthly rates (per population) of single-vehicle, night-time crashes from 2000 to 2012	ITS
Colchero et al., 2020 [41]	Drivers, passengers, and pedestrians aged 15 years or older	Fixed and random sobriety checkpoints in Mexico City, Mexico (2003) (specific alcohol policy)	Not external, pre-intervention. Legal BAC of 0.4 g/l previously in place (specific alcohol policy)	Monthly rates (per population) of crashes resulting in fatalities from 1998 to 2016	ITS
Davenport et al., 2021 [42]	Drivers, passengers, and pedestrians aged 15 years or older	Reduction of legal BAC to zero in Uruguay (2016) (specific alcohol policy)	External. Continued legal BAC of 0.3 g/l in Chile (specific alcohol policy)	Monthly number of crashes resulting in moderate/severe injuries and fatalities at any time, night-time, and weekends from 2013 to 2017	CITS
Desapriya et al., 2009 [43]	Drivers, passengers, and pedestrians aged 15 years or older	Deregulation of alcohol production and sales in Japan (1994) (general alcohol policy)	Not external, pre-intervention. Tight regulation of alcohol production and sales (general alcohol policy)	Annual rates (per population) of single-vehicle, nigh-time and day-time crashes resulting in injuries and fatalities from 1985 to 2001	BA
Desapriya et al., 2012 [44]	Drivers, passengers, and pedestrians aged 15 years or older	Deregulation of alcohol production and sales in Japan (1994) (general alcohol policy)	Not external, pre-intervention. Tight regulation of alcohol production and sales (general alcohol policy)	Annual rates (per population) of age- and sex-specific crashes resulting in fatalities from 1985 to 2002	BA
French et al., 2009 [45]	Motorcyclists aged 15 years and older	Road-safety laws and specific alcohol policies (i.e., the reduction of legal BAC to 0.8 g/l, YZTL, and ALR) across the continental USA. As observed between 1990 to 2005 (gradual and heterogeneous roll-out)	Not external, pre-intervention. Alcohol policy not specified	Annual rates (per population) of crashes resulting in injuries and fatalities from 1990 to 2005	ITS
Han et al., 2015 [46]	Drivers, passengers, and pedestrians aged 15 years or older	Introduction of a large number of off-sale alcohol outlets in Lubbock, TX (USA) (2009) (general alcohol policy)	External. Off-sale outlets already in place in Bryan – College Station, TX (USA) (general alcohol policy)	Monthly rates (per population) of crashes and single-vehicle, night-time crashes from 2007 to 2012	CITS
Humphreys et al., 2020 [47]	Drivers, passengers, and pedestrians aged 15 years or older	Subsidized rideshare trips from bars and restaurants (9 PM to 2 AM) in Evesham and Voorhees, NJ, USA, between 2015 to 2018 (specific alcohol policy)	External. Non-participating municipalities in NJ, USA. Alcohol policy not specified	Annual rates (per roadway kilometers) of time of day-specific crashes resulting in injuries from 2010 to 2018	CITS
Jiang, Livingston, and Manton, 2015 [48]	Drivers, passengers, and pedestrians aged 17–39	RBT and lowering the MLDA to 18 years in four Australian states. Gradual and heterogeneous roll-out from 1970 to 1988 (specific and general alcohol policies)	Not external, pre-intervention. No RBT and MLDA of 21 (general alcohol policy)	Annual number of age-specific crashes resulting in fatalities from 1951 to 2010	ITS
Jiang, Livingston, and Room, 2015 [49]	Drivers, passengers, and pedestrians aged 15 years or older	CSBL and RBT implemented in all Australian states. Gradual roll-out from 1970 to 1988 (specific alcohol policy)	Not external, pre-intervention. Alcohol policy not specified	Annual rates (per population) of age- and sex-specific crashes resulting in fatalities from 1924 to 2006	ITS
Mader and Zick, 2014 [50]	Non-motorists aged 15 years or older	Road-safety laws and specific alcohol policy (reduction of legal BAC to 0.8 g/l) across all USA states. As observed between 1999 to 2009 (gradual and heterogeneous roll-out)	Not external, pre-intervention. Legal BAC of 1.0 g/l (specific alcohol policy)	Annual rates (per population) of crashes resulting in fatalities from 1999 to 2009	ITS
Miller et al., 2004 [51]	Drivers, passengers, and pedestrians aged 15 years or older	CBT + YZTL, aggressive media campaign, and booze buses in New Zealand. Gradual and heterogeneous roll-out from 1993 to 1996 (specific alcohol policies)	Not external, pre-intervention. External for booze buses. Legal BAC of 0.8 g/l (all ages) (specific alcohol policy)	Quarterly or annual number of night-time crashes resulting in serious injuries or fatalities from 1987 to 2001	ITS
Nghiem et al., 2016 [52]	Drivers, passengers, and pedestrians aged 15 years or older	Various specific alcohol policies taking place in Qld., Australia (e.g., legal BAC, RBT, and YZTL). Gradual roll-out from 1968 to 2004	Not external, pre-intervention. Alcohol policy not specified	Annual number of crashes (per vehicles registered) resulting in fatalities from 1958 to 2007	ITS
Notrica et al., 2020 [53]	Drivers and passengers 16 years and older	Driver-related laws, general and specific alcohol policies (e.g., MLDA and DUI penalties) across all USA states. As observed between 1999 to 2015 (gradual and heterogeneous roll-out)	Not external, pre-intervention. Alcohol policy not specified	Annual rates (per population) of age-specific crashes resulting in fatalities from 1999 to 2015	ITS
Pridemore et al., 2013 [54]	Drivers, passengers, and pedestrians aged 15 years or older	A comprehensive and stringent regulation on the production and sale of alcohol products in Russia (2006) (general alcohol policy)	Not external, pre-intervention. Alcohol policy not specified—likely less stringent	Monthly number of sex-specific crashes resulting in fatalities from 2000 to 2010	ITS
Sebego et al., 2014 [55]	Drivers, passengers, and pedestrians aged 15 years or older	Alcohol levies, increased fines and penalties for DUIA in Botswana. Gradual roll-out from 2008 to 2010 (specific and general alcohol policies)	Not external, pre-intervention. Lower fines and penalties for DUIA (specific alcohol policy)	Monthly rates (per fuel volume sales) of crashes, crashes resulting in fatalities, and single-vehicle night-time crashes resulting in fatalities from 2004 to 2011	ITS
Sen, 2016 [56]	Drivers, passengers, and pedestrians aged 15 years or older	Varying degrees of deregulated retail access to alcohol in three Canadian provinces (Alta., B.C., and Que.). As observed between 1998 to 2010 (general alcohol policy)	External. Largely regulated alcohol markets (i.e., government-run stores) in three Canadian provinces (Man., Ont., Sask.). As observed between 1998 to 2010 (general alcohol policy)	Annual rates (per population) of crashes resulting in injuries and fatalities from 1998 to 2010	CITS
Stockwell et al., 2001 [57]	Drivers, passengers, and pedestrians aged 15 years or older	Alcohol levy in NT, Australia (1992), gradually funding treatment and prevention programs (general alcohol policy)	Not external, pre-intervention. Increased enforcement of liquor laws (general alcohol policy). Minor crash injuries (control series)	Quarterly rates (per driver population) of night-time crashes resulting in serious injuries and all fatal road crashes from 1984 to 1996	ITS
Trolldal, 2005 [58]	Drivers, passengers, and pedestrians aged 15 years or older	Privatization of the retail sale of alcohol in Alta., Canada. Gradual roll-out from 1985 to 1994 (general alcohol policy)	External. Canada as a whole—except for Alta. Alcohol policy not specified	Annual rates (per population) of crashes resulting in fatalities from 1950 to 1998	CITS
Vingilis et al., 2007 [59]	Drivers, passengers, and pedestrians aged 15 years or older	Extended alcohol sales and service hours to 2 AM in Ont., Canada (1996) (general alcohol policy)	Not external, pre-intervention. Hours for alcohol sales and services up to 1 AM (general alcohol policy)	Monthly number of motor-vehicle night-time crashes resulting in injuries (trauma admissions) from 1992 to 1999	ITS
Voas, 2008 [60]	Drivers aged 15 years or older	Sobriety checkpoints in Charlottesville, VA, USA, during 1984 (specific alcohol policy)	Not external, pre-intervention. All crashes in Charlottesville and VA, USA (contrast measures). Alcohol policy not specified	Monthly percentages of all crashes resulting in night-time crashes from 1981 to 1984	CITS

*Abbreviations. BAC* blood alcohol concentration, *RBT* random breath testing, *MLDA* minimum legal drinking age, *CBT* compulsory breath testing, *YZTL* youth zero-tolerance law, *CSBL* compulsory seat-belt law, *DUIA* driving under the influence of alcohol, *ALR* administrative license revocation

*Abbreviations for places.* *VA* Virginia, *B.C*. British Columbia, *Ont* Ontario, *TX* Texas, *USA* United States. *Alta* Alberta, *Que* Quebec, *Sask* Saskatchewan, *Man* Manitoba. *NT* Northern Territory, *Qld* Queensland, *NJ* New Jersey

*Abbreviations for study designs*. *BA* uncontrolled before-after study *ITS* interrupted time series, *CITS* controlled interrupted time series

*Notes *"Gradual roll-out" refers to the gradual introduction of an intervention over time

"Heterogeneous roll-out" refers to the partial introduction of an intervention across territories

"As observed" refers to the status of an intervention within a given period in a particular territory (i.e., changed, never had, or always had the intervention)

The included studies evaluated alcohol policies implemented in the United States (*n* = 7), Australia (*n* = 4), Canada (*n* = 4), and Japan (*n* = 2), with a single study each conducted in Botswana, Brazil, Mexico, New Zealand, Norway, the Russian Federation, and Uruguay. We identified a total of 33 alcohol policies evaluated, which we defined as general (*n* = 12) [43, 44, 46, 48, 53, 54, 56–59] and specific (*n* = 21) [37–42, 45, 47–53, 55, 60].

Based on the information obtained from the studies, we categorized general alcohol policies into:

- *Alcohol regulation*: shrinkage of the market-driven production and sales of alcohol by tightening state regulations controlling alcohol availability and consumption (e.g., banning of alcohol production and sale without a license, prohibition of alcohol sales at public places, taxes on the production, sales, or consumption of alcohol products) [54, 55, 57].
- *Alcohol deregulation*: expansion of the market-driven production and sales of alcohol by increasing alcohol availability and lowering restrictions (e.g., the extension of drinking hours or increase in off-sale alcohol outlets) [43, 44, 46, 56, 58, 59].
- *Minimum-legal drinking age*: legal age when an individual can purchase alcoholic beverages [48, 53].
- *Adult responsibility*: laws related to adult responsibility for underage drinking, such as social host laws [53].

We categorized specific alcohol policies as follows:

- *BAC limit*: introducing or modifying a BAC limit above which motor vehicle driving is prohibited. We also included laws related to zero tolerance and specific BAC limits for young people [37–39, 42, 45, 50, 52, 53].
- *Sobriety checkpoints*: roadside breathalyzer tests, fixed or mobile, random or not, for detecting DUIA cases, which can be complemented by media campaigns or alcohol buses [41, 48, 49, 51, 52, 60].
- *Penalties for driving under the influence of alcohol*: can include license or vehicle confiscation, referral to remedial programs, processing fees, or other punitive means [40, 45, 53, 55].
- *Alcohol in transport*: laws punishing possession and use of alcohol in transport, for instance, in the case of passengers of non-commercial vehicles [53].
- *Community awareness*: programs to raise community awareness on the risk of DUIA [52].
- *Subsidized ridesharing*: programs promoting rideshare (i.e., an alternative transportation means) to reduce DUIA [47].

Five studies complementarily evaluated non-alcohol-related road safety measures. In the United States, French et al. [45], Mader and Zick [50], and Notrica et al. [53] assessed the specific effects of several alcohol policies (e.g., lowering the minimum legal drinking age, the reduction of the BAC limit, and introducing penalties for DUIA, among others) and road safety policies, such as graduated driver license programs for adolescent drivers, maximum legal speed limits, seat belt laws, and universal helmet laws. In Australia, Jiang et al. assessed the joint effects of the introduction of compulsory seat belt legislation and random sobriety checks [49]; and Nghiem et al., among other specific alcohol policies, assessed the Safe4Life program: a road safety strategy that gave priority to raising awareness on DUIA and drugs [52].

Most of the comparators in the included studies were based on data for the same jurisdiction (i.e., not external) before the alcohol policy went into effect (i.e., pre-intervention) (*n* = 19) [37–41, 43–45, 48–55, 57, 59, 60]. There were five instances in which researchers relied on an external comparator [42, 46, 47, 56, 58]. For example, Davenport et al. used data from Chile as a comparator to assess a reduction in the BAC limit in Uruguay [42]. According to Davenport et al., Chile had a continuous BAC limit during the study period [42]. Similarly, in Canada, Sen compared varying degrees of alcohol deregulation in the provinces of Alberta, British Columbia, and Quebec to largely regulated alcohol markets in the provinces of Manitoba, Ontario, and Saskatchewan [56].

Regarding the type of intervention in the comparison condition, most studies did not specify the type of alcohol policy during the pre-intervention period or in the external jurisdiction (*n* = 8) [45, 47, 49, 52–54, 58, 60]. Studies that reported an alcohol policy for the comparison condition referred to a BAC limit (*n* = 7) [37, 39, 41, 42, 47, 50, 51] or the presence of stricter alcohol regulation (*n* = 5) [43, 44, 56, 57, 59]. The existence of lower penalties for DUIA (*n* = 2) [40, 55], alcohol deregulation [46], and a higher minimum-legal drinking age were also included as comparators [48].

As for the type of road traffic outcome, studies considered fatal crashes (*n* = 19) [37–39, 41–45, 48–58], injury crashes (*n* = 9) [37, 38, 42, 44, 45, 47, 56, 57, 59], or crashes (*n* = 4) [40, 46, 55, 60]. The type, time of day, and day of the week of crashes were not specified for all outcomes. We assumed these outcomes referred to all types of crashes, regardless of the time or day of the week [37, 41–50, 52–58]. When this information was provided, studies reported analyzing night-time crashes (*n* = 12) [38–40, 42, 44, 46, 47, 51, 55, 57, 59, 60], single-vehicle crashes (*n* = 6) [38–40, 44, 46, 55], weekend crashes (*n* = 2) [38, 42], or day-time crashes (*n* = 2) [44, 47]. On the time interval between outcome data points, most of the studies relied on annual (*n* = 12) [38, 43–45, 47–50, 52, 53, 56, 58] or monthly data (*n* = 10) [37, 39–42, 46, 54, 55, 59, 60], with a few using quarterly (i.e., 3-month interval) data (*n* = 2) [51, 57]. The median number of time units was 49.5, with a minimum of 9 annual data points in Humphreys et al. [47], to a maximum of 228 monthly data points in Colchero et al. [41].

When exploring the reasons for not using DUIA data, most studies did not report why DUIA data were missing (*n* = 12) [45, 46, 48–54, 56, 58, 59]. The remainder claimed that data on DUIA were unavailable, difficult to obtain, or partially available (*n* = 8) [37, 38, 41, 42, 44, 55, 57, 60] or stated that data on DUIA were unreliable (*n* = 4) [39, 40, 43, 47].

In terms of study design, we identified three studies with an uncontrolled before-and-after design [38, 43, 44], 15 studies with an interrupted time series design [37, 39–41, 45, 48–55, 57, 59], and six with a controlled interrupted time-series design [42, 46, 47, 56, 58, 60]. The most used time-series method was the autoregressive integrated moving average (ARIMA) model [37, 46, 48, 49, 51, 54, 55, 57–60]. Further details on the characteristics of included studies are provided in Additional file 1: Appendix 2.

### Risk of bias in studies

**Fig. 2:**
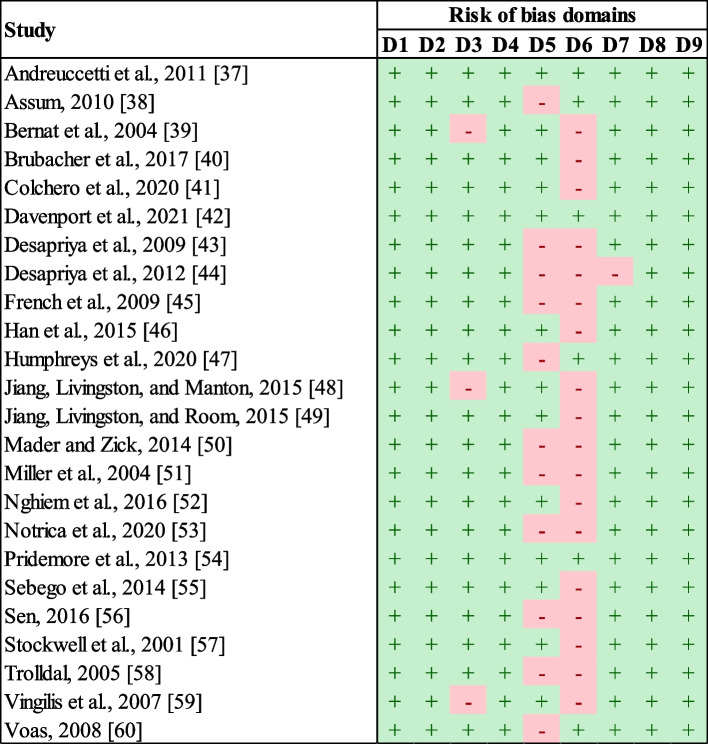
Summary of the risk of bias assessment *Notes*. Risk of bias domains were as follows: D1, study question; D2, eligibility criteria and study population; D3, representativeness of study participants; D4, participants' enrollment; D5, sample size; D6, description and implementation of the intervention; D7, outcomes measures; D8, statistical analysis; and D9, multiple outcome measures. Studies having a low risk of bias in a specific domain were represented by a green plus sign. Those studies judged as having some concern for bias in a specific domain were represented by a red negative sign

A summary of the RoB assessment is provided in Fig. 2. Almost half of the studies (*n* = 11) did not have the minimum sample size required for a well-powered time series analysis (i.e., 50 units of time). The median sample size for this subset of studies was 17 units of time [38, 43–45, 47, 50, 51, 53, 56, 58, 60]. Most of these studies used annual data [38, 43–45, 47, 50, 53, 56, 58]. Six studies had a low RoB when assessing the description and implementation of the intervention [37, 38, 42, 47, 54, 60]. Some studies assumed a homogeneous roll-out of alcohol policies over time, but the implementation process was poorly described to justify such an assumption adequately [41, 43, 44, 46]. There were five studies in which the insufficient detail of the interventions was coupled with the risk of co-intervention, being these other alcohol policies or road safety interventions [45, 50, 52, 53, 57]. A subgroup of these studies did not report the implementation dates of alcohol policies, the extension of the post-intervention period or the degree of co-interventions remained unclear [45, 50, 53]. Eight studies acknowledged that the evaluation of alcohol policies was not consistently applied over time or to the whole study population [39, 40, 48, 49, 51, 55, 56, 58]. Of these studies, Sebego et al. [55] assessed alcohol policies that were introduced along with road safety co-interventions, and Jiang et al. [49] could only report the combined effects of an alcohol policy and a road safety co-intervention. Finally, Vingilis et al. [59] acknowledged concerns over attributing study results to road safety co-interventions.

### Results of studies and synthesis

**Fig. 3:**
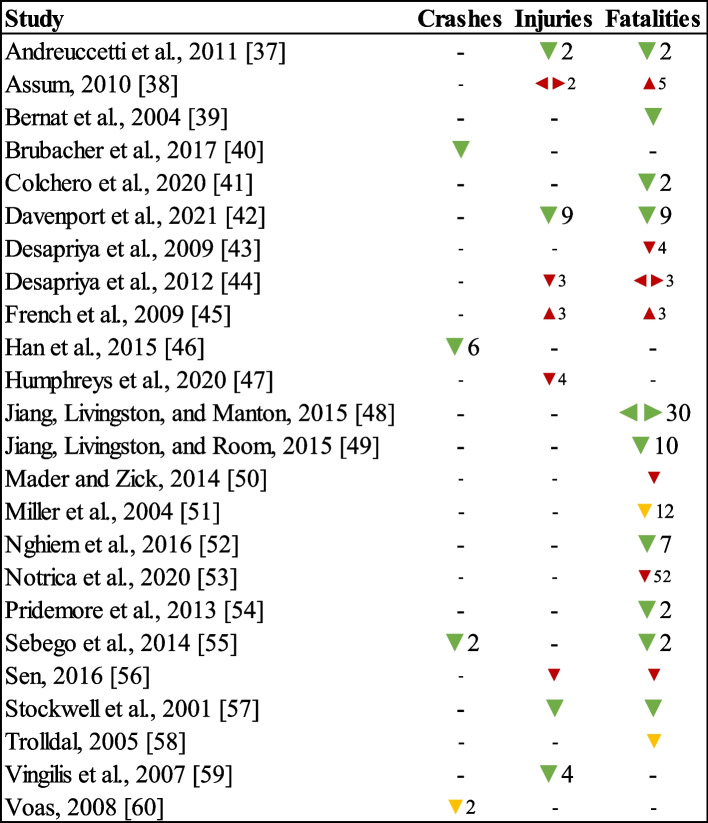
Effect direction plot summarizing the direction of road traffic outcomes in studies assessing alcohol policies. [37–60]. *Notes*. Effect direction: upward arrow ▲ = negative health impact (e.g., increase in fatalities); downward arrow ▼ = positive health impact (e.g., decrease in injuries); sideways arrow ◄► = no change/mixed effects/conflicting findings. Units of time in the study: large arrow  > 50; medium arrow  25-49; small arrow  < 24. Numbers beside each arrow represent the number of road traffic outcomes summarized, and no number appears in cases where only one outcome was evaluated. In Miller et al. (2004), we classified the actual outcome (severe injuries or fatalities) as fatalities

We identified a total of 188 road traffic outcomes grouped into road traffic crashes (*n* = 11), injuries (*n* = 29), and fatalities (*n* = 148). The effect direction plot summarizes the direction of the effect estimates for road traffic outcomes at the study level (Fig. 3).

For road traffic crashes, all five studies reported a positive effect direction (i.e., a decrease in crashes) (*P-*value for the binomial probability test = 0.125). Regarding road traffic injuries, 7 of 9 studies had a positive effect direction, one with a negative effect direction, and one with an unclear effect direction (*P-*value for the binomial probability test = 0.070). Concerning road traffic fatalities, 15 studies had a positive effect direction, two with a negative effect direction, and two with an unclear effect direction (*P-*value for the binomial probability test = 0.002). According to the binomial probability tests, there was sufficient evidence of an association between alcohol policies and positive effect directions for traffic fatalities.

The effect direction plots summarizing the direction of road traffic outcomes at the study level, grouped by potential methodological modifiers, are displayed in Additional file 1: Appendix 3, Figs. 1a through 4a. Data and the *P*-value for the binomial probability tests synthesizing effect directions across studies per potential methodological modifiers are shown in Additional file 1: Appendix 3, Tables 1a through 4a. We did not have enough information on the type of road traffic crash to consider this variable as a possible cause of heterogeneity among study results (i.e., only six studies reported such information, all of which used single-vehicle crashes).

Subgroup analyses initially showed evidence for an association between potential methodological modifiers and positive effect directions for road traffic fatalities. In the subgroup of 12 studies that assessed road traffic fatalities following the implementation of specific alcohol policies, ten studies reported a positive effect direction, and two had a negative effect direction (*P-*value for the binomial probability test = 0.039) (Table 2a). Thirteen of seventeen studies assessing road traffic fatalities not occurring during the night-time (i.e., any other crashes) had a positive effect direction, one had a negative effect direction, and three had unclear effect directions (*P-*value for the binomial probability test = 0.002) (Table 4a). There was no evidence for an association between traffic fatalities following the implementation of general alcohol policies or those occurring during the night-time and positive effect directions (Tables 1a and 3a) Fisher's exact test results suggest no statistically significant differences in the proportion of studies reporting positive effect directions between the strata of the potential methodological modifiers (i.e., general vs. specific policies and night-time crashes vs. any other crashes). These results are detailed in Tables 5a and 6a of Additional file  1: Appendix 3.

## Discussion

### General interpretation of the results

We included twenty-four eligible studies. Regarding objective 1, when DUIA data were missing, most studies assessed road traffic fatalities following the introduction of specific alcohol policies (e.g., the modification of a BAC limit). These studies generally relied on night-time crashes as a surrogate for an objective measure of DUIA. Notably, the rationale for the surrogate measure was frequently not reported. These studies were mainly conducted in high-income countries, particularly USA, and employed uncontrolled interrupted time series designs. Regarding objective 2, we found evidence for an association between alcohol policies and decreased traffic fatalities, with no associations found in the case of road traffic crashes or injuries. Subgroup analyses for studies having a positive direction of effect on road traffic fatalities by type of alcohol policy or temporal information on road traffic crashes found no statistically significant differences between groups.

### Limitations of the evidence included in the review

The evidence included in the review had critical limitations on sample size and the description and implementation of the interventions. Half of the included studies did not meet the minimum requirement of 50 units of time for well-powered time series analyses. Only two acknowledged problems related to statistical power and the length of the study period [50, 58]. Generally, studies using annual data had such limitations. While we recognize that an adequate assessment of power should consider a set of factors (such as sample size per time period, anticipated effect size, and location of the intervention in the time series, among others) [61, 62], none of the reviewed studies discussed these elements.

Regarding the description and implementation of the intervention, two key questions were not satisfactorily answered in the studies: the specific components of the policies and their consistent implementation over time and space. We found insufficient information on the components of general alcohol policies. For instance, it was unclear if alcohol deregulation jointly considered a decrease in alcohol-specific taxes and an increase in off-sale alcohol outlets. The heterogeneous or interrupted implementations further complicated policy evaluations. The capacity needed for implementation and enforcement was often overlooked, which is needed to judge implementation consistency. Other studies have also highlighted the challenges in alcohol policy evaluation due to their complex implementation [13, 63]. Concomitant interventions that could provide alternative explanations for observed changes attributed to alcohol policies were identified. These complexities affect model construction and estimation in time series analyses. Caution is warranted in interpreting syntheses results, as three-quarters of the reviewed studies were assessed with concerns for bias in this critical domain.

### Limitations of the review processes

To ensure transparency and quality in our research process, we registered the review protocol in PROSPERO, and adhered to the recommendations of the Cochrane Collaboration, the PRISMA statement, and the SWiM guidelines for systematic reviews without meta-analyses. However, our review process is subject to several potential biases. Our scope was limited to studies published in peer-reviewed journals in English or French from 2000 onwards, potentially excluding relevant literature from non-conventional sources, other languages, or before 2000, resulting in selection bias. As the searches for our systematic review may have been outdated, we conducted an updated MEDLINE search of studies published between November 2021 and April 2023 but found no eligible studies (Additional file 1: Appendix 1).

Additionally, changes to the protocol, detailed in Additional file 1: Appendix 1, may have introduced bias. We broadened the search strategy to compensate for lowering the information sources. We used the QAT-BA [33] instead of the Risk of Bias In Non-randomised Studies – of Interventions (ROBINS-I) as the tool for RoB [64]. The ROBINS-I was not used due to its complexity, high assessor burden, assessor dependency, and requirements for intensive training and supervision that exceeded the resources allocated to the study [65–67].

The results of our scoping review should be considered exploratory and provisional, given the limitations of our synthesis method and the small sample of included studies. Vote counting based on the direction of effects lacks information on the magnitude of effects, does not account for differences in the relative sizes of the studies, and does not provide a formal assessment of the certainty of our findings [32]. The binomial probability test for effect direction synthesis should be carefully interpreted due to methodological concerns (e.g., issues of statistical power, the well-known limitations of *P*-values and significance testing, and restrictive underlying assumptions) [36]. The binomial probability test results aid in interpreting the overall pattern of effect direction [36]. The small sample of included studies limited our ability to explore potential effect modifiers in detail.

### Implications for practice, policy, and future research

The availability and reliability of DUIA data (e.g., BAC) pose challenges for road safety professionals, policymakers, and researchers [12, 20, 22]. Surrogate measures like night-time crashes might seem reasonable alternatives when DUIA data are missing. However, caution is needed when interpreting road traffic outcomes associated with alcohol policies in such cases. In our scoping review, a minority of non-randomized studies provided sufficient information for outcomes assessment, utilizing uncontrolled designs with monthly counts of night-time crashes. DUIA data were missing in these studies because of their unavailability or unreliability. Notably, half of the studies did not report the type, time of day, day of the week crashes occurred, or the reasons for using surrogate measures. This lack of transparency hinders the proper evaluation of alcohol policies. Greater efforts should be made to report outcomes assessed in detail to inform evidence-based interventions.

Our exploratory synthesis found an association between alcohol policies and reduced road traffic fatalities when DUIA data are missing. However, as highlighted in the previous paragraph, this finding must be interpreted cautiously. Rather than providing certainty, the quality of the reviewed evidence makes us reflect on the characteristics that a study with missing DUIA data should have. We believe such a study should have access to more granular data (monthly or quarterly) and pay attention to the number of events per unit of time to have sufficient statistical power to detect differences. The intervention and its level of implementation should be well-defined, and this information should be incorporated into data modeling. The potential for co-intervention and confounders should be accounted [68]. Such a study should use a controlled design, comparing outcomes with those of other jurisdictions with similar characteristics where the intervention has not occurred.

Regarding future research directions, we found that studies from LMICs are urgently needed to provide more direct evidence on using surrogate measures for DUIA data. We know from Brazil, Botswana, Mexico, and Russia studies that access to more granular data is possible in these locations. Complementarily, we also found that the evidence coming from developed countries is particularly weighted towards the USA. On the other hand, studying single-vehicle or weekend crashes requires further investigation as surrogate measures. Finally, results from other reviews where DUIA data were available should be formally compared with the results of our review to compare the proportions of outcomes with a positive direction of effect. Such a comparison could provide an estimate to confirm or recalibrate the associations found in studies where DUIA data were missing.

## Supplementary Information

**Additional file1.**

## Data Availability

The data that support the findings of this study are openly available in Hardvard Dataverse at https://doi.org/10.7910/DVN/NNATZM [69].

## Abbreviations

BAC: Blood alcohol concentration
DUIA: Driving under the influence of alcohol
GBD: Global Burden of Diseases, Injuries, and Risk Factors Study
JINM: José Ignacio Nazif-Munoz
JJ: Junon Joseph
LMIC: Low- and middle-income countries
PM: Pablo Martínez
PICOS: Population, Intervention, Comparator, Outcomes, and Study Design
PRISMA: Preferred Reporting Items for Systematic Reviews and Meta-Analyses
PROSPERO: Prospective Register of Systematic Reviews
QAT-BA: Quality Assessment Tool for Before-After (Pre-Post) Studies With No Control RoB: Group Risk of Bias
SWiM: Synthesis Without Meta-analysis
WHO: World Health Organization
