# Supplementary material for: The absence of data on driving under the influence of alcohol in road traffic studies: a scoping review of non-randomized studies with vote counting based on the direction of effects of alcohol policies
Source: Subst Abuse Treat Prev Policy. 2023 Jul 28;18:46. doi: 10.1186/s13011-023-00553-y (PMC10375679; doi:10.1186/s13011-023-00553-y)
Supplement: Supplementary file 1 — Additional file1. [file 13011_2023_553_MOESM1_ESM.docx]

**Appendices to:**

**The absence of data on drinking under the influence of alcohol in road traffic studies: a scoping review of non-randomized studies with vote counting based on the direction of effects of alcohol policies**

Pablo Martínez^a,b, c^^[[1]](#footnote-1)^

Junon Joseph^a^

José Ignacio Nazif-Munoz^a,b,c^

^a^Faculté de médecine et des sciences de la santé, Université de Sherbrooke, 150, place Charles-Le Moyne, Longueuil, Québec J4K A08, Canada.

^b^Centre de recherche Charles-Le Moyne—Saguenay-Lac-Saint-Jean sur les innovations en santé (CR-CSIS), 150, place Charles-Le Moyne, Longueuil, Québec J4K A08, Canada.

^c^Institut universitaire sur les dépendances. 950 rue de Louvain Est, Montréal, Québec H2M 2E8, Canada

**Appendix 1**

**Amendments to the protocol**

- Eligibility criteria:
  - Interventions: Different alcohol policies were defined, and the focus was not exclusively on specific alcohol policies (i.e., directly deterring drinking and driving). General alcohol policies were also included.
  - Comparator: We also included control conditions with an active/other alcohol policy.
  - Study design: A explicit definition of quasi-experimental studies was provided, including study designs initially presented as observational. Systematic reviews, meta-analyses, and editorials were relocated to report/publication eligibility criteria.
- Information sources: Originally, we considered the following databases to conduct searches for relevant literature: Embase, MEDLINE, and PsycINFO databases via Ovid, the Web of Sciences Core Collection, SafetyLit, Criminal Justice Database (ProQuest), and the Transport Research International Documentation (TRID) databases.
- Search strategy: The piloted search strategy as written in the protocol, which included free-text words related to alcohol, policies, and road safety outcomes, was further modified and simplified to broaden search results. We compared the performance of our original search strategy (i.e., alcohol AND policy AND traffic AND [crashes OR injuries OR deaths OR fatalities], including the powerful 'apply related words' function) with the search strategy that we finally used in our systematic review. Had we used the original search strategy we would have retrieved around 1,200 search results – almost half of the search results that our definitive search strategy retrieved. The link for the original search strategy: <https://web-p-ebscohost-com.ezproxy.usherbrooke.ca/ehost/search/advanced?vid=13&sid=a3ca48c6-98d2-4eb0-a30b-983ef6e97503%40redis>.
- We acknowledge that the latest search date reported in our scoping review is November 2021; as of April 2023, this is more than a year old. To compare with an up-to-date search, in April 2023, we conducted a quick and controlled search in MEDLINE (through EBSCOhost) (The link to the updated search strategy: <https://web-s-ebscohost-com.ezproxy.usherbrooke.ca/ehost/resultsadvanced?vid=6&sid=be8439dd-418f-45e8-99c8-f7431d46ae8d%40redis&bquery=%22alcohol%22+AND+%22traffic+crashes%22&bdata=JmRiPW1uaCZjbGkwPURUMSZjbHYwPTIwMjExMS0wMDAwMDEmY2xpMT1MQTk5JmNsdjE9RW5nJTdlRnJlJnR5cGU9MSZzZWFyY2hNb2RlPUFuZCZzaXRlPWVob3N0LWxpdmU%3d>). Between November 2021 and April 2023, the search result yielded 22 new records. After examination of the title and abstract, we evaluated two full-text reports for eligibility – i.e., studies assessing the impact of alcohol policies on road traffic outcomes (Lenk et al., 2023; Straßgütl & Evers, 2022). Both studies had DUIA data, so they would not have been included in our review.
- Data items: The data items section was reframed and expanded, with a detailed account of data items. We also made explicit assumptions when information was not available. The original data items considered in the protocol were characteristics of publications and policies studied and the study population, methodology, and relevant findings. In contrast, the new data items are characteristics, population, intervention, comparator, outcomes, study design, and study results.
- Risk of bias (RoB) assessment: We used the Study Quality Assessment Tool for Before-After (Pre-Post) Studies With No Control Group (QAT-BA) instead of the Risk of Bias In Non-randomised Studies – of Interventions (ROBINS-I) (Sterne et al., 2016). We recognize that ROBINS-I is a rigorous and recommended tool for RoB assessment in non-randomized studies (Sterne et al., 2016); this was the reason for its inclusion in the original protocol. However, we considered using QAT-BA, which is relatively simple to apply. ROBINS-I, on the contrary, has been a tool characterized by its complexity, high assessor burden, assessor dependency, usually poorly applied, and requiring intensive training and supervision that exceeded the resources allocated to the study (Igelström et al., 2021; Jeyaraman et al., 2020; Jeyaraman et al., 2021).
- Synthesis methods: Prespecified group analyses according to world region or country income level were not conducted due to lack of variability (i.e., most of studies were conducted in high-income countries). The so-called "narrative synthesis" strategy was abandoned, instead a Synthesis Without Meta-analysis (SWiM) approach was taken, as per Campbell et al (2020). Vote counting based on direction of effect was used as a synthesis method.

**Appendix 2**

**Supplementary information for Table 1**

- Age was not reported and assumed to be 15 years or older in: (Andreuccetti et al., 2019; Assum, 2010; Colchero et al., 2020; Davenport et al., 2021; Desapriya et al., 2009, 2012; Han et al., 2015; Humphreys et al., 2020; Mader & Zick, 2014; Miller et al., 2004; Nghiem et al., 2016; Sebego et al., 2014; Stockwell et al., 2001; Trolldal, 2005; Vingilis et al., 2007; Voas, 2008).
- Type of road user was not reported and assumed to be drivers, passengers, cyclists, and pedestrians in: (Andreuccetti et al., 2019; Colchero et al., 2020; Davenport et al., 2021; Desapriya et al., 2009, 2012; Han et al., 2015; Humphreys et al., 2020; Jiang, Livingston, & Room, 2015; Miller et al., 2004; Nghiem et al., 2016; Sen, 2016; Trolldal, 2005; Vingilis et al., 2007).
- According to Bernat et al. (2004), other fatal traffic crashes were used as a control series and alcohol license revocation laws (a specific alcohol policies) were also introduced during the study period.
- In Brubacher et al. (2017), the driver population demographics were reported at the patrol level (Brubacher et al., 2017).
- In Desapriya et al. (2012), the population was defined as teenagers and adults, without specifying age.
- French et al. (2009) assessed the effects of road-safety laws (i.e., universal helmet law, mandatory rider education program, and the speed limit on rural interstates), and the stated specific alcohol policies. The highest observed legal blood alcohol concentration (BAC) limit of 1.2 g/l was assigned to states that did not have an explicit legal BAC. Alaska, Hawaii, and the District of Columbia were excluded from the study.
- In Jiang et al. (2015), the joint effects of random breath testing (RBT) and compulsory seat-belt law (CSBL) in the 1970s were explored. RBT is a specific alcohol policy. CSBL is a road safety policy.
- In Mader and Zick (2014), non-motorists were defined as walking pedestrians, bicyclists, individuals in a wheelchair or motorized personal conveyances, skateboarders, and others. The study assessed the effects of road-safety laws (e.g., driver cellphone ban, graduated driver license systems, bike helmets, etc.), and the specific alcohol policy specified in Table 1.
- According to Miller et al. (2004), the implementation dates were 1993 for CBT + youth zero-tolerance law (YZTL), 1995 for the media campaign, and 1996 for booze buses; booze buses were added for about one-third of New Zealand. In this study, regions where booze buses were not implemented were used as controls
- In Nghiem et al. (2016), the various specific alcohol policies taking place were: the lowering of the legal BAC to 1.0 g/l in 1968, 0.8 g/l in 1974, and 0.5 g/l in 1982; RBT in 1988; zero BAC limit for learner drivers and young drivers in 1991; expansion of the RBT in 1997; and a road safety strategy giving priority to addressing driving under the influence of drugs and alcohol in 2004.
- Notrica et al. (2020) assessed the effects of driver-related laws (e.g., CSBL, mature population driver license, cellphone ban, etc.), including specific alcohol policies (legal BAC, driving under the influence of alcohol laws, underage alcohol laws, social host laws, alcohol in transportation laws).
- In Pridemore et al. (2013), the general alcohol policy assessed banned alcohol production and sale without a license, alcohol production regulated by the government, excise stamp for alcohol products, and prohibition of alcohol sales at certain public places, among other measures.
- According to Sebego et al. (2014), alcohol levy was implemented in October 2008 and increased in November 2010. A comprehensive Road Traffic Act went into effect in April 2009.
- According to Sen et al. (2016), between 1993 to 2002, British Columbia was considered a largely regulated alcohol market. From 2003 onwards changed to a partially deregulated alcohol market (intervention).
- According to Stockwell et al. (2001), various policies were implemented during the study period, before and after the intervention. Price increases funded more treatment and prevention programs, and BAC of 0.05 g/l was implemented in December 1994. In the same study, night-time crashes occurring on Thursdays, Fridays, and Saturdays resulting in injuries requiring hospital treatment were considered.
- Studies using time-series designs employed the following methods:
  - Autorregresive integrated moving average (Andreuccetti et al., 2019; Han et al., 2015; Jiang, Livingston, & Manton, 2015; Jiang, Livingston, & Room, 2015; Miller et al., 2004; Pridemore et al., 2013; Sebego et al., 2014; Stockwell et al., 2001; Trolldal, 2005; Vingilis et al., 2007; Voas, 2008).
  - Difference-in-differences (Humphreys et al., 2020).
  - Fixed-effects model (French et al., 2009).
  - Generalized autoregressive time-series modeling (Notrica et al., 2020).
  - Mixed-effects model (Bernat et al., 2004; Brubacher et al., 2017; Mader & Zick, 2014).
  - Ordinary least squares and generalized least squares time-series (Sen, 2016).
  - State-space time-series using seemingly unrelated time-series equations (Nghiem et al., 2016).
  - Synthetic controls (Davenport et al., 2021).

**Appendix 3**

**Supplementary Tables and Figures**

**Figure 1a. Effect direction plot summarizing the direction of road traffic outcomes in studies assessing general alcohol policies.**

*Notes.*

Effect direction: upward arrow ▲= negative health impact (e.g., increase in fatalities); downward arrow ▼= positive health impact (e.g., decrease in injuries); sideways arrow ◄►= no change/mixed effects/conflicting findings.

Time units in the study: large arrow ▲ >50; medium arrow ▲ 25-49; small arrow ▲ <24.

**Table 1a. Summary of the direction of effects for road traffic outcomes in studies assessing general alcohol policies.**

|  | **Crashes** | **Injuries** | **Fatalities** |
| --- | --- | --- | --- |
| **Positive** | 1 | 4 | 6 |
| **Negative** | 0 | 0 | 1 |
| **Total (unclear, not counted)** | 1 | 4 | 7 |
| **Total (unclear counted)** | 1 | 4 | 8 |
| **Binomial probability test's *P*-value** | 1.000 | 0.125 | 0.125 |

**Figure 2a. Effect direction plot summarizing the direction of road traffic outcomes in studies assessing specific alcohol policies.**

*Notes.*

Effect direction: upward arrow ▲= negative health impact (e.g., increase in fatalities); downward arrow ▼= positive health impact (e.g., decrease in injuries); sideways arrow ◄►= no change/mixed effects/conflicting findings.

Time units in the study: large arrow ▲ >50; medium arrow ▲ 25-49; small arrow ▲ <24.

In Miller et al. (2004), we classified the actual outcome (severe injuries or fatalities) as fatalities.

**Table 2a. Summary of the direction of effects for road traffic outcomes in studies assessing specific alcohol policies.**

| **Total** | **Crashes** | **Injuries** | **Fatalities** |
| --- | --- | --- | --- |
| **Positive** | 2 | 3 | 10 |
| **Negative** | 0 | 1 | 2 |
| **Total (unclear, not counted)** | 2 | 4 | 12 |
| **Total (unclear counted)** | 2 | 5 | 12 |
| **Binomial probability test's *P*-value** | 0.500 | 0.625 | 0.039 |

**Figure 3a. Effect direction plot summarizing the direction of road traffic outcomes in studies assessing alcohol policies that used night-time crashes.**

*Notes.*

Effect direction: upward arrow ▲= negative health impact (e.g., increase in fatalities); downward arrow ▼= positive health impact (e.g., decrease in injuries); sideways arrow ◄►= no change/mixed effects/conflicting findings.

Time units in the study: large arrow ▲ >50; medium arrow ▲ 25-49; small arrow ▲ <24.

In Miller et al. (2004), we classified the actual outcome (severe injuries or fatalities) as fatalities.

**Table 3a. Summary of the direction of effects for road traffic outcomes in studies assessing alcohol policies that used night-time crashes.**

| **Total** | **Crashes** | **Injuries** | **Fatalities** |
| --- | --- | --- | --- |
| **Positive** | 3 | 4 | 5 |
| **Negative** | 0 | 1 | 1 |
| **Total (unclear, not counted)** | 3 | 5 | 6 |
| **Total (unclear counted)** | 3 | 5 | 6 |
| **Binomial probability test's *P*-value** | 0.250 | 0.375 | 0.219 |

**Figure 4. Effect direction plot summarizing the direction of road traffic outcomes in studies assessing alcohol policies that used any other type of crashes.**

*Notes.*

Effect direction: upward arrow ▲= negative health impact (e.g., increase in fatalities); downward arrow ▼= positive health impact (e.g., decrease in injuries); sideways arrow ◄►= no change/mixed effects/conflicting findings.

Time units in the study: large arrow ▲ >50; medium arrow ▲ 25-49; small arrow ▲ <24.

**Table 4a. Summary of the direction of effects for road traffic outcomes in studies assessing alcohol policies that used any other type of crashes.**

| **Total** | **Crashes** | **Injuries** | **Fatalities** |
| --- | --- | --- | --- |
| **Positive** | 1 | 6 | 13 |
| **Negative** | 0 | 1 | 1 |
| **Total (unclear, not counted)** | 1 | 7 | 14 |
| **Total (unclear counted)** | 2 | 7 | 17 |
| **Binomial probability test's *P*-value** | 1.000 | 0.125 | 0.002 |

**Table 5a. Positive and negative direction of effects for road traffic outcomes between studies assessing general and specific alcohol policies.**

| **Total** | *Positive* | *Negative* |
| --- | --- | --- |
| **Crashes** |  |  |
| *General policy* | 1 | 0 |
| *Specific policy* | 2 | 0 |
| **Two-tailed Fisher's exact *P*-value** |  | 1.000 |
| **Injuries** |  |  |
| *General policy* | 4 | 0 |
| *Specific policy* | 3 | 1 |
| **Two-tailed Fisher's exact *P*-value** |  | 1.000 |
| **Fatalities** |  |  |
| *General policy* | 6 | 1 |
| *Specific policy* | 10 | 2 |
| **Two-tailed Fisher's exact *P*-value** |  | 1.000 |

**Table 6a. Positive and negative direction of effects for road traffic outcomes between studies using night-time or any other type of crashes.**

| **Total** | *Positive* | *Negative* |
| --- | --- | --- |
| **Crashes** |  |  |
| *Night-time crashes* | 3 | 0 |
| *Any other type of crashes* | 1 | 0 |
| **Two-tailed Fisher's exact *P*-value** |  | 1.000 |
| **Injuries** |  |  |
| *Night-time crashes* | 4 | 1 |
| *Any other type of crashes* | 6 | 1 |
| **Two-tailed Fisher's exact *P*-value** |  | 1.000 |
| **Fatalities** |  |  |
| *Night-time crashes* | 5 | 1 |
| *Any other type of crashes* | 13 | 1 |
| **Two-tailed Fisher's exact *P*-value** |  | 0.521 |

**References**

Andreuccetti, G., Leyton, V., Carvalho, H. B., Sinagawa, D. M., Bombana, H. S., Ponce, J. C., Allen, K. A., Vecino-Ortiz, A. I., & Hyder, A. A. (2019). Drink driving and speeding in Sao Paulo, Brazil: Empirical cross-sectional study (2015–2018). *BMJ Open*, *9*(8), e030294. https://doi.org/10.1136/bmjopen-2019-030294

Assum, T. (2010). Reduction of the blood alcohol concentration limit in Norway—Effects on knowledge, behavior and accidents. *Accident Analysis & Prevention*, *42*(6), 1523–1530. https://doi.org/10.1016/j.aap.2010.03.002

Bernat, D. H., Dunsmuir, W. T. M., & Wagenaar, A. C. (2004). Effects of lowering the legal BAC to 0.08 on single-vehicle-nighttime fatal traffic crashes in 19 jurisdictions. *Accident; Analysis and Prevention*, *36*(6), 1089–1097. https://doi.org/10.1016/j.aap.2004.04.001

Brubacher, J., Chan, H., Erdelyi, S., Asbridge, M., & Schuurman, N. (2017). Factors Predicting Local Effectiveness of Impaired Driving Laws, British Columbia, Canada. *Journal of Studies on Alcohol and Drugs*, *78*(6), 899–909. https://doi.org/10.15288/jsad.2017.78.899

Campbell, M., McKenzie, J. E., Sowden, A., Katikireddi, S. V., Brennan, S. E., Ellis, S., Hartmann-Boyce, J., Ryan, R., Shepperd, S., Thomas, J., Welch, V., & Thomson, H. (2020). Synthesis without meta-analysis (SWiM) in systematic reviews: Reporting guideline. BMJ, 368, l6890. https://doi.org/10.1136/bmj.l6890

Colchero, M. A., Guerrero-López, C. M., Quiroz-Reyes, J. A., & Bautista-Arredondo, S. (2020). Did "Conduce Sin Alcohol" a Program that Monitors Breath Alcohol Concentration Limits for Driving in Mexico City Have an Effect on Traffic-Related Deaths? *Prevention Science: The Official Journal of the Society for Prevention Research*, *21*(7), 979–984. https://doi.org/10.1007/s11121-020-01133-3

Davenport, S., Robbins, M., Cerdá, M., Rivera-Aguirre, A., & Kilmer, B. (2021). Assessment of the impact of implementation of a zero blood alcohol concentration law in Uruguay on moderate/severe injury and fatal crashes: A quasi-experimental study. *Addiction (Abingdon, England)*, *116*(5), 1054–1062. https://doi.org/10.1111/add.15231

Desapriya, E., Fujiwara, T., Dutt, N., Arason, N., & Pike, I. (2012). Impact of the 1994 alcohol production and sales deregulation policy on traffic crashes and fatalities in Japan. *Asia-Pacific Journal of Public Health*, *24*(5), 776–785. https://doi.org/10.1177/1010539511404398

Desapriya, E., Fujiwara, T., Scime, G., Sasges, D., Pike, I., & Shimizu, S. (2009). Are 1994 alcohol production and the sales deregulation policy in Japan associated with increased road traffic fatalities among adult and teenage males and females in Japan? *Nihon Arukoru Yakubutsu Igakkai Zasshi = Japanese Journal of Alcohol Studies & Drug Dependence*, *44*(5), 569–578.

French, M. T., Gumus, G., & Homer, J. F. (2009). Public policies and motorcycle safety. *Journal of Health Economics*, *28*(4), 831–838. https://doi.org/10.1016/j.jhealeco.2009.05.002

Han, D., Shipp, E. M., & Gorman, D. M. (2015). Evaluating the effects of a large increase in off-sale alcohol outlets on motor vehicle crashes: A time-series analysis. *International Journal of Injury Control and Safety Promotion*, *22*(4), 320–327. https://doi.org/10.1080/17457300.2014.908223

Humphreys, D. K., Degli Esposti, M., Williams, F. M., Kondo, M. C., & Morrison, C. (2020). Assessing the impact of a local community subsidised rideshare programme on road traffic injuries: An evaluation of the Evesham Saving Lives programme. *Injury Prevention: Journal of the International Society for Child and Adolescent Injury Prevention*, injuryprev–2020–043728. https://doi.org/10.1136/injuryprev-2020-043728

Igelström, E., Campbell, M., Craig, P., & Katikireddi, S. V. (2021). Cochrane's risk of bias tool for non-randomized studies (ROBINS-I) is frequently misapplied: A methodological systematic review. *Journal of Clinical Epidemiology*, *140*, 22–32. https://doi.org/10.1016/j.jclinepi.2021.08.022

Jeyaraman, M. M., Rabbani, R., Copstein, L., Robson, R. C., Al-Yousif, N., Pollock, M., Xia, J., Balijepalli, C., Hofer, K., Mansour, S., Fazeli, M. S., Ansari, M. T., Tricco, A. C., & Abou-Setta, A. M. (2020). Methodologically rigorous risk of bias tools for non-randomized studies had low reliability and high evaluator burden. *Journal of Clinical Epidemiology*, *128*, 140–147. https://doi.org/10.1016/j.jclinepi.2020.09.033

Jeyaraman, M. M., Robson, R. C., Copstein, L., Al-Yousif, N., Pollock, M., Xia, J., Balijepalli, C., Hofer, K., Mansour, S., Fazeli, M. S., Ansari, M. T., Tricco, A. C., Rabbani, R., & Abou-Setta, A. M. (2021). Customized guidance/training improved the psychometric properties of methodologically rigorous risk of bias instruments for non-randomized studies. *Journal of Clinical Epidemiology*, *136*, 157–167. https://doi.org/10.1016/j.jclinepi.2021.04.017

Jiang, H., Livingston, M., & Manton, E. (2015). The effects of random breath testing and lowering the minimum legal drinking age on traffic fatalities in Australian states. *Injury Prevention: Journal of the International Society for Child and Adolescent Injury Prevention*, *21*(2), 77–83. https://doi.org/10.1136/injuryprev-2014-041303

Jiang, H., Livingston, M., & Room, R. (2015). Alcohol consumption and fatal injuries in Australia before and after major traffic safety initiatives: A time series analysis. *Alcoholism, Clinical and Experimental Research*, *39*(1), 175–183. https://doi.org/10.1111/acer.12609

Lenk, K. M., Toomey, T. L., MacLehose, R. F., Scholz, N., Schriemer, D., Nelson, T. F., Delehanty, E., Bosma, L. M., & Gloppen, K. (2023). Place of last drink enforcement: Effects on alcohol-related traffic crashes. *Alcoholism, clinical and experimental research*, 47(2), 406–413. https://doi.org/10.1111/acer.15001

Mader, E. M., & Zick, C. D. (2014). Active transportation: Do current traffic safety policies protect non-motorists? *Accident Analysis & Prevention*, *67*, 7–13. https://doi.org/10.1016/j.aap.2014.01.022

Miller, T., Blewden, M., & Zhang, J. (2004). Cost savings from a sustained compulsory breath testing and media campaign in New Zealand. *Accident; Analysis and Prevention*, *36*(5), 783–794. https://doi.org/10.1016/j.aap.2003.07.003

Nghiem, S., Commandeur, J. J. F., & Connelly, L. B. (2016). Determinants of road traffic safety: New evidence from Australia using state-space analysis. *Accident; Analysis and Prevention*, *94*, 65–72. https://doi.org/10.1016/j.aap.2016.05.012

Notrica, D. M., Sayrs, L. W., Krishna, N., Rowe, D., Jaroszewski, D. E., & McMahon, L. E. (2020). The impact of state laws on motor vehicle fatality rates, 1999-2015. *The Journal of Trauma and Acute Care Surgery*, *88*(6), 760–769. https://doi.org/10.1097/TA.0000000000002686

Pridemore, W. A., Chamlin, M. B., Kaylen, M. T., & Andreev, E. (2013). The impact of a national alcohol policy on deaths due to transport accidents in Russia. *Addiction (Abingdon, England)*, *108*(12), 2112–2118. https://doi.org/10.1111/add.12311

Sebego, M., Naumann, R. B., Rudd, R. A., Voetsch, K., Dellinger, A. M., & Ndlovu, C. (2014). The impact of alcohol and road traffic policies on crash rates in Botswana, 2004-2011: A time-series analysis. *Accident; Analysis and Prevention*, *70*, 33–39. https://doi.org/10.1016/j.aap.2014.02.017

Sen, A. (2016). Is Retail Alcohol Deregulation Correlated with More Crime and Traffic Injuries? Evidence from Canadian Provinces. *Canadian Journal of Criminology and Criminal Justice*, *58*(2), 251–286. https://doi.org/10.3138/cjccj.20160304

Sterne, J. A., Hernán, M. A., Reeves, B. C., Savović, J., Berkman, N. D., Viswanathan, M., Henry, D., Altman, D. G., Ansari, M. T., Boutron, I., Carpenter, J. R., Chan, A.-W., Churchill, R., Deeks, J. J., Hróbjartsson, A., Kirkham, J., Jüni, P., Loke, Y. K., Pigott, T. D., … Higgins, J. P. (2016). ROBINS-I: A tool for assessing risk of bias in non-randomised studies of interventions. BMJ, 355, i4919. https://doi.org/10.1136/bmj.i4919

Straßgütl, L., & Evers, C. (2022). Long-term effects of the German zero tolerance law for novice drivers. *Journal of Safety Research*, 80, 46-53. https://doi.org/10.1016/j.jsr.2021.11.003

Stockwell, T., Chikritzhs, T., Hendrie, D., Fordham, R., Ying, F., Phillips, M., Cronin, J., & O'reilly, B. (2001). The public health and safety benefits of the Northern Territory's Living with Alcohol programme. *Drug and Alcohol Review*, *20*(2), 167–180. https://doi.org/10.1080/09595230124886

Trolldal, B. (2005). An investigation of the effect of privatization of retail sales of alcohol on consumption and traffic accidents in Alberta, Canada. *Addiction (Abingdon, England)*, *100*(5), 662–671. https://doi.org/10.1111/j.1360-0443.2005.01049.x

Vingilis, E., McLeod, A. I., Stoduto, G., Seeley, J., & Mann, R. E. (2007). Impact of extended drinking hours in Ontario on motor-vehicle collision and non-motor-vehicle collision injuries. *Journal of Studies on Alcohol and Drugs*, *68*(6), 905–911. https://doi.org/10.15288/jsad.2007.68.905

Voas, R. B. (2008). A new look at NHTSA's evaluation of the 1984 Charlottesville Sobriety Checkpoint Program: Implications for current checkpoint issues. *Traffic Injury Prevention*, *9*(1), 22–30. https://doi.org/10.1080/15389580701682114

1. **Corresponding author:** email: [Pablo.Alberto.Martinez.Diaz@USherbrooke.ca](mailto:Pablo.Alberto.Martinez.Diaz@USherbrooke.ca); full postal address: 150, place Charles-Le Moyne, Longueuil, QC J4K 0A8, Canada. [↑](#footnote-ref-1)
